# A comparison of practices, distributions and determinants of birth attendance in two divisions with highest and lowest skilled delivery attendance in Bangladesh

**DOI:** 10.1186/s12884-018-1770-9

**Published:** 2018-05-02

**Authors:** Gulam Muhammed Al Kibria, Vanessa Burrowes, Allysha Choudhury, Atia Sharmeen, Swagata Ghosh, Anna Kalbarczyk

**Affiliations:** 1 Department of Epidemiology and Public Health, University of Maryland School of Medicine, Baltimore, MD 21201, USA; 2 Department of International Health, Johns Hopkins Bloomberg School of Public Health, Baltimore, MD 21205, USA; 3 Department of Epidemiology, Johns Hopkins Bloomberg School of Public Health, Baltimore, MD 21205, USA; 4 School of Community Health and Policy, Morgan State University, Baltimore, MD 21251, USA; 5 Nova Scotia Health Authority, Nova Scotia, Canada

**Keywords:** Delivery attendance, Skilled birth attendants, Khulna, Sylhet, Bangladesh

## Abstract

**Background:**

Delivery by skilled birth attendants (SBAs) is strongly recommended to reduce maternal and neonatal mortality. The percentage of births attended by SBAs is low in Bangladesh (42% in 2014), though this rate varies widely by divisions, with the highest 58% in Khulna and only 27% in Sylhet. Comparing and critically analyzing the practices, distributions and determinants of delivery attendance in two divisions with the highest and lowest SBA attendance could help to understand the differences and to employ the findings of the high-performing division to the low-performing division.

**Methods:**

The 7th Bangladesh Demographic and Health Survey (BDHS 2014) data were analyzed. After reporting the types of delivery attendants, logistic regression analyses were applied to calculate the odds ratios of determinants of deliveries attended by SBAs.

**Results:**

SBAs attended 225 (58.6%) and 128 (27.4%) deliveries in Khulna and Sylhet, respectively. Khulna had higher birth attendance by qualified doctors (42.5%, *n* = 163) than Sylhet (15.8%, *n* = 74). Sylhet had higher attendance by traditional attendants (60.8%, *n* = 285) than Khulna (33.7%, *n* = 129). In both regions, attendance by community skilled birth attendants (CSBAs) was very low (< 1%). Khulna had higher percentages of women with higher education level, husbands’ higher education, antenatal care (ANC) visits by SBAs, and higher wealth quintiles than Sylhet.

In multivariable analyses, higher education level (adjusted odds ratio (AOR): 8.4; 95% confidence interval (CI): 1.9–36.7), ANC visits (AOR: 3.6; 95% CI: 2.0–6.5), family planning workers’ visit (AOR: 3.0; 95% CI: 1.6–5.4), and belonging to richer (AOR: 2.6; 95% CI: 1.4–5.1) or richest (AOR: 3.8; 95% CI: 1.9–7.6) household wealth quintiles had significant positive associations with deliveries by SBAs in Sylhet. Similarly, ANC visits (AOR: 2.5; 95% CI: 1.4–4.6) and higher wealth quintile (AOR: 4.7; 95% CI: 1.9–11.5) were positive predictors in Khulna.

**Conclusions:**

The higher proportion of educated women and their husbands, wealth status and ANC visits were associated with higher SBA utilization in Khulna compared to Sylhet. Improvement of socioeconomic status, increasing birth attendant awareness programs, providing ANC services, and family-planning workers’ visits could increase the proportion of SBA-attended deliveries in Sylhet Division. CSBA program should be re-evaluated for both divisions.

## Plain English summary

Delivery by skilled birth attendants (SBAs) is recommended to reduce maternal and neonatal mortality. The nation of Bangladesh has a low proportion of births attended by SBAs, though there is a large variation of this by divisions within the country. To better understand the distribution and determinants of SBA usage, we compared the lowest and highest ranking divisions of SBA usage (Sylhet and Khulna, respectively) by analyzing Bangladesh Demographic and Health Survey (BDHS 2014) data. We found that Khulna had higher birth attendance by qualified doctors than Sylhet, and low community skilled birth attendant (CSBA) usage in both divisions. Higher education level, antenatal care by a skilled provider, visits from a family planning worker, and high household wealth quintile, were significantly associated with delivery by SBAs in Sylhet. However, only antenatal care by skilled providers and higher wealth quintile were significantly associated with SBA delivery in Khulna. In conclusion, higher educational level of women and their husbands, higher wealth status, and receiving antenatal checkups appear to be essential factors that contribute to higher SBA utilization in Khulna compared to Sylhet. The government and local NGOs should incorporate these factors into CSBA programs and interventions to increase SBA usage of these divisions.

## Background

From 1990 to 2015, Bangladesh experienced a substantial reduction in maternal and neonatal mortality. In this period, the estimated maternal mortality ratio (MMR) declined from 550 to 176 per 100,000 live births (LB) [1], and the neonatal mortality rate (NMR) decreased from 63 to 23 per 1000 LB [2]. The United Nations’ (UN) Millennium Development Goals (MDGs) targeted to reduce the MMR by three-fourths and child mortality by two-thirds within the year 2015 compared to their 1990 levels. Although Bangladesh achieved the child mortality target, this country was not able to meet the maternal mortality target of the MDGs [1, 2]. Then in 2015, the health-related goal (Goal 3) of the UN’s Sustainable Development Goals (SDGs) set targets to reduce MMR and NMR to less than 70 per 100,000 LB and 12 per 1000 LB, respectively, by the year 2030 [3]. The NMR target was explicitly set due to the slower reduction of NMR compared to under-five mortality rate [2]. From 1990 to 2015, the estimated annual rate of reduction (ARR) of MMR and NMR were 4.5% and 3.9%, respectively in Bangladesh [1, 2]; these ARRs need to be increased to 6.0% and 4.5%, respectively to achieve the targets of SDGs.

Skilled birth attendants (SBAs) can manage most obstetrics complications during childbirth that could reduce maternal and neonatal mortality substatially [4]. An SBA is a certified health professional who has been educated and trained in the skills needed to manage cases of simple pregnancies, child-births, the immediate postnatal period. They can detect, manage or refer complications in women and newborns. SBA includes qualified doctors, nurses, midwives, family welfare visitors (FWVs) and community skilled birth attendants (CSBAs). As they can manage most obstetric complications, SBAs are strongly recommended for all types of deliveries [4]. However, the latest Bangladesh Demographic and Health Survey 2014 (BDHS 2014) estimated that only 42% of the births were attended by SBAs in Bangladesh [5]. To achieve the MMR and NMR targets, the proportion of SBA- attended deliveries needs to be increased. Given the lower SBA prevalence during births in Bangladesh, reasons for the lack of SBA usage should be investigated to better understand how to promote SBAs in all parts of the country.

**Fig. 1:**
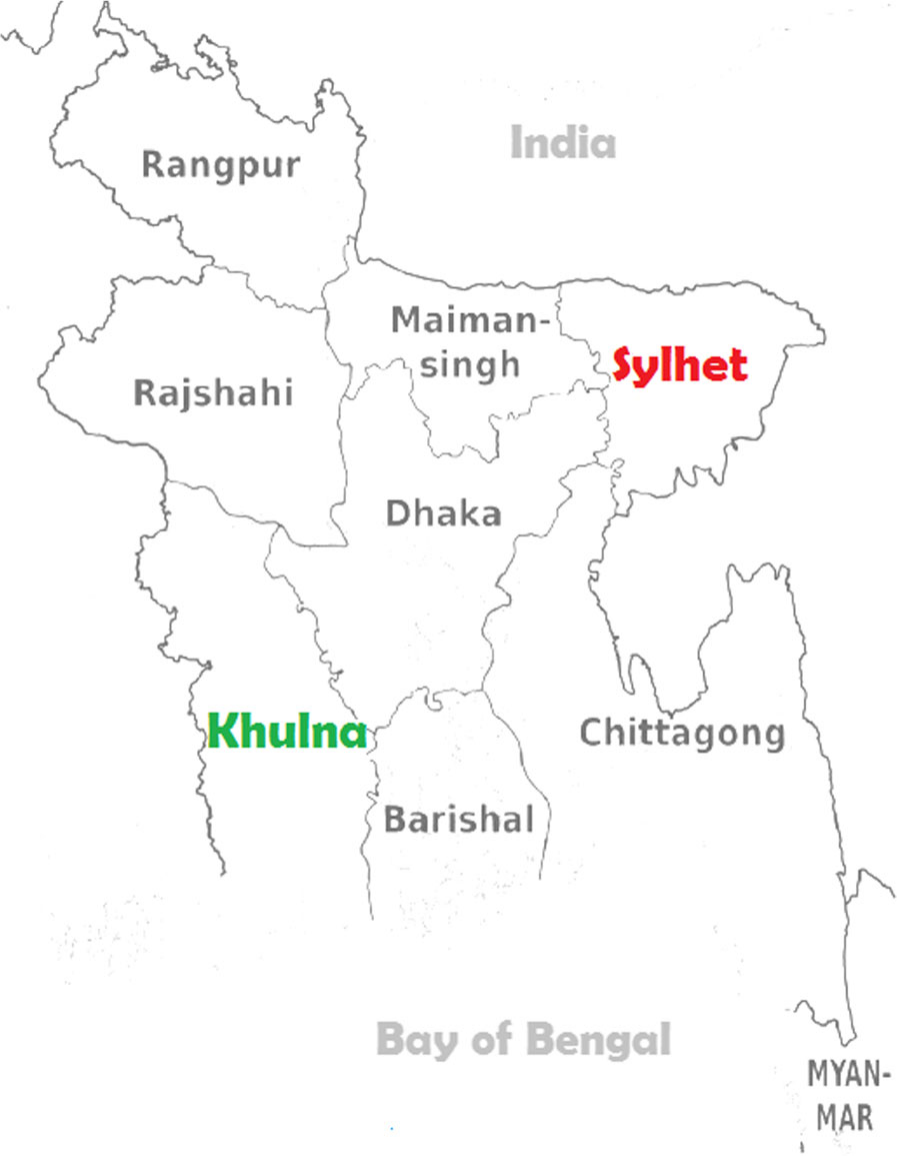
Map of Bangladesh *(Adapted from Bangladesh Demographic and Health Survey 2014* [5])

Bangladesh is a developing country in South Asia with a land mass of approximately 144,000 square-kilometers. This country is divided into several divisions, which is the largest administrative unit of the country [6]. The BDHS 2014 concluded that there are wide divisional variations in health indicators and utilization of health care services including deliveries attended by SBAs in the country. Approximately 58% of the deliveries were attended by SBAs in Khulna Division, while this rate was only 27% in Sylhet [5]. Khulna Division is located in south-western part of the country and Sylhet division in the north-eastern region (Fig. 1) [6]. The headquarters of these two divisions are approximately 250 km away from the Capital City, Dhaka and 450 km away from each other. Ordering from higher to lower-level, both of the divisions are divided into several administrative districts, sub-districts, and unions, respectively. According to the latest census, the estimated population of Sylhet and Khulna are 1 and 1.5 million, respectively [7]. The majority of the population in these two divisions lives on agriculture. Khulna region is predominantly a coastal region while Sylhet Division has some hilly areas in addition to wetlands [6]. The BDHS 2014 also found that the health indicators of the Sylhet Division are the poorest among the divisions in this country [5].

Overall, this country has a pluralistic health system. In addition to public facilities, the private facilities and non-government organizations also play a major role in the health system of this country. The Government of Bangladesh provides free maternal and child health-care services in all regions of the country through health facilities such as the medical college, district, and sub-district hospitals. The families have to purchase some additional drugs which are not available in the facilities [8]. Despite having similar levels of health care services from the Government, the health indicators are worst in Sylhet Division including the proportion of SBA-attended deliveries compared to those of other divisions [5].

The percentage of SBA-attended deliveries in Khulna surpassed the target of the Health, Population and Nutrition Sector Development Program (HPNSDP), which set a target to provide skilled attendance to at least 50% of the deliveries within 2016 [9]. However, estimated SBA attendance was almost half of the target in Sylhet. These regional differences in SBA utilization may be the primary reason for the overall low prevalence and point to the need for promoting SBA-attendance in low-performing divisions. Comparison and analyses of the practices, determinants and distribution of delivery attendance of these two divisions could help to understand the differences and the potential sectors to be prioritized in the low-performing division or would contribute to employ ‘findings’ of the high-performing division to the division with lower performance.

Earlier studies demonstrated that educational status of women and husbands, maternal occupation, wealth status, exposure to mass media, receiving antenatal care (ANC), receiving a visit from family planning workers (FPWs), transportation issues, complications related to delivery, and place of residence (rural or urban areas) influences care seeking behavior for delivery [10–18]. The aims of the current study are to investigate the differences in distribution of these selected factors, and to examine their association with delivery attendance in addition to the practices of child births in the two selected divisions with highest and lowest SBA attendance in Bangladesh by analyzing BDHS 2014 data.

## Methods

### Ethics statement

The demographic and health survey (DHS) data are available for academic use upon approval; ethical approval for our study was not required. The official approval to use the data was obtained from the ICF International, Rockville, Maryland, USA in August 2016.

### Data source

The BDHS 2014 was the 7th DHS from Bangladesh. Mitra and Associates, a private research organization in Bangladesh, conducted this survey from June to November 2014. The details of the survey including survey design, methodologies, findings, and questionnaires are available elsewhere [5].

To conduct this interview, BDHS 2014 used a household questionnaire, a women’s questionnaire, and a community questionnaire. The women’s questionnaire was used to interview ever-married women aged 15–49 years of age. One hundred and sixty-four field workers were recruited and trained to conduct the oral interview [5].

Women provided information on their demographic characteristics, reproductive history, family planning behavior, antenatal care, delivery, postnatal, and newborn care, breastfeeding and infant feeding practices, child immunizations and illnesses, marriage, fertility preferences, husband’s background and respondent’s work, and awareness of AIDS and other sexually transmitted infections [5].

### Sample design and coverage

To make the BDHS 2014 sample nationally representative, the survey used a sampling frame from the list of enumeration areas (EAs) of the 2011 Population and Housing Census of the People’s Republic of Bangladesh [5].

In brief, BDHS 2014 used a two-stage stratified sample of households and selected 600 EAs with probability proportional to the EA size, with 207 EAs in urban areas and 393 in rural areas in the first stage. To provide a sampling frame for the second-stage selection of households, the survey carried out a complete household listing operation in all selected EAs and selected a systematic sample of 30 households on average per EA to provide statistically reliable estimates of key demographic and health variables. This was conducted for the country as a whole, for urban and rural areas separately, and for each of the seven divisions in the second stage. By this method, the survey selected 18,000 households and 18,000 ever-married women were expected to be interviewed [5].

For weighting of the distribution of urban-rural households, BDHS 2014 employed the urban-rural distribution in the 2011 population census of the country. Adjustment of the sample weights reflected a modified urban-rural household distribution. In this way, any significant differences in the overall survey indicators were not expected to occur [5].

Initially, the survey selected and interviewed 17,989 and 17,300 households, respectively. These interviewed households had 18,245 ever-married women of reproductive age (i.e., 15– 49 years); 17,863 women completed interviews with a response rate of 98%. Rural and urban areas had a similar response rate [5].

### Participants

To minimize recall bias, among the 17,863 interviewed women, only the deliveries occurring in the last 3 years were included to report the number of deliveries attended by SBAs. The total number of weighted deliveries in the Khulna and Sylhet were 384 and 469, respectively.

### Outcome

Women responded about the attendants of their last child-birth. To incorporate deliveries attended by SBAs in the analysis, we used the SBA definition provided by the BDHS 2014; qualified doctors, nurses, midwives, paramedics, FWVs, and CSBAs were defined as skilled attendants. Medical assistants, medical assistants or sub-assistant community medical officers (SACMOs), community health care providers (CHCPs), traditional birth attendants (TBAs), unqualified doctors such as village doctors or untrainned doctors, relatives, neighbors, and others were defined as unskilled attendants [5].

### Statistical analyses

Potential factors were selected based on published reports and data structure of the BDHS 2014. For these analyses, Stata 13.0 (Stata Corp, College Station, TX) was used [19]. In Stata, the ‘svy’ command was used to allow for the adjustments of the cluster sampling design used in the BDHS 2014 and to estimate weighted frequency for all explanatory variables.

**Fig. 2:**
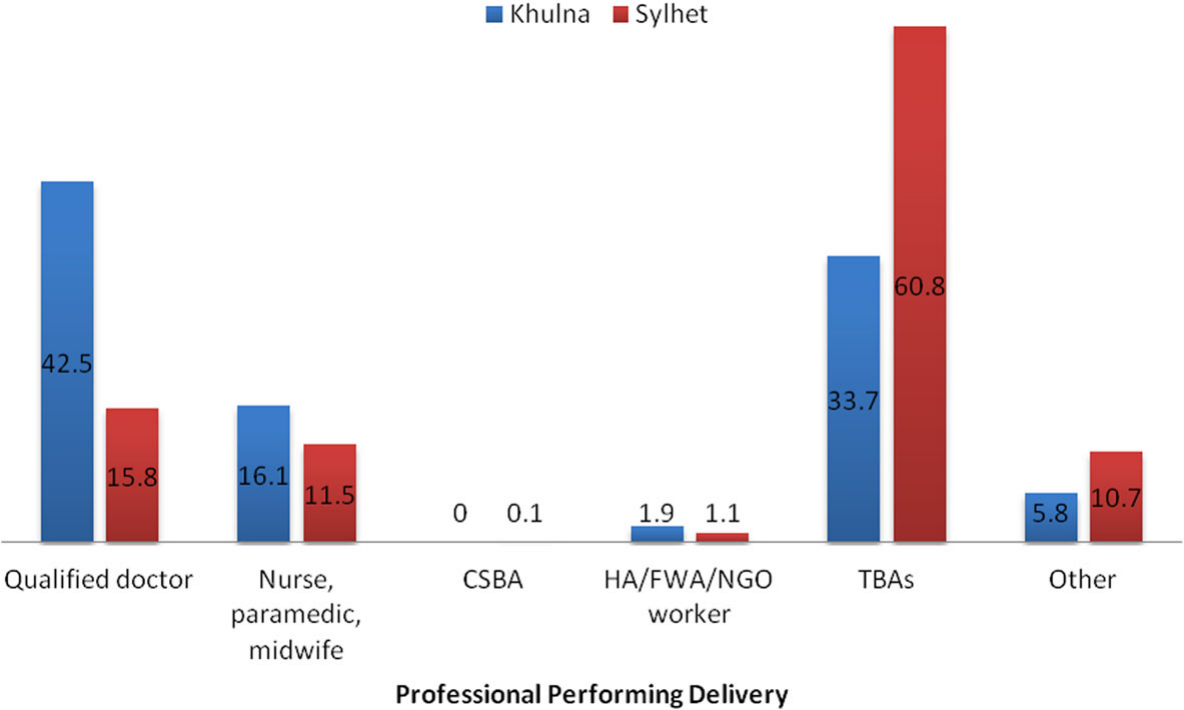
Percentage of deliveries by birth attendants (CSBA: Community skilled birth attendants; HA: Health assistants; FWA: Family welfare assistant; NGO: Non-government organization; TBA: Traditional birth attendants)

The weighted percentage of the deliveries according to the types of birth attendants (Fig. 2) was calculated. Women were compared by weighted distributions of selected variables. Lastly, logistic regression analyses were applied to calculate the crude (unadjusted) odds ratios (CORs) and adjusted odds ratios (AORs) of SBA attendance for selected variables. Odds ratios (ORs) were calculated with 95% confidence intervals (CIs) and significance level. To aid in the logistic regression analyses, continuous and discrete variables were converted into categorical variables. To check for the presence of multi-collinearity among variables before incorporating into the multivariable adjustment, variance inflation factors (VIFs) were assessed after calculating the CORs. Variables with a predetermined significance level (*p* < 0.2) were included in multivariable analysis. This significance level is considered to be sufficient for adjustment of confounders [20]. Principal component analysis of basic housing construction materials, sources of water, sanitation facilities, electricity, and household belongings was employed to construct wealth index score for each household. The wealth status was stratified into 5 categories: poorer, poorest, middle, richer and richest.

## Results

Figure 2 shows the weighted percentage of the deliveries according to birth attendants. A total of 384 and 469 deliveries from Khulna and Sylhet were included in the analysis, respectively. The percentage of deliveries attended by SBAs in Khulna was 58.6% (*n* = 225). This percentage was 27.4% (*n* = 128) in Sylhet. In Khulna, qualified doctors attended 42.5% (*n* = 163) deliveries, while in Sylhet it was only 15.8% (*n* = 74). In Khulna, TBAs attended 33.7% (*n* = 129) deliveries, whereas this percentage was almost doubled in Sylhet, 60.8% (*n* = 285). Sylhet also had a higher proportion of child-births attended by unqualified doctors (i.e., untrainned or village doctors) or relatives (other unskilled attendants). In both the Khulna and Sylhet Divisions, the percentage of deliveries attended by CSBA was extremely low.

Table 1 describes the comparison of child-birth in the two study divisions by the distribution of birth attendants. After applying the weighted frequency distribution, Khulna had a higher SBA attendance rate compared to Sylhet in all categories and sub-categories. Khulna also had a greater percentage of women with college level education or higher than Sylhet, with 12.1% (*n* = 47) and 4.8% (*n* = 22), respectively. The percentage for women without any formal education was higher in Sylhet (23.4%, *n* = 110) compared to Khulna (7.1%, *n* = 27). In both divisions, highly educated women and women with highly educated husbands had a higher proportion of deliveries attended by SBAs. In Khulna, 82.2% (*n* = 316) women received ANC services, as compared to 58.5% (*n* = 275) in Sylhet. In both divisions, women with ANC services during pregnancy were more likely to use SBAs than women who did not receive ANC services. Women from higher wealth quintiles had higher proportions of skilled attendants during deliveries. Rural women of both divisions tended to use SBAs lower than their urban counterparts.

**Table 1:** Weighted percentage distribution of deliveries by skilled birth attendants (SBAs), with selected background characteristics, in Khulna and Sylhet Division, Bangladesh

Characteristics	Khulna Division’s SBA attendance			Sylhet Division’s SBA attendance		
	No (%)^a^	Yes (%)^a^	Total (n)^b^	No (%)^a^	Yes (%)^a^	Total (n)^b^
Current age (years)						
15–24	42.0	58.0	216	71.6	28.4	245
25–34	41.4	58.6	154	69.7	30.3	182
35–49	32.1	67.9	14	90.6	9.4	42
Parity						
1	35.4	64.6	152	64.6	35.4	152
≥ 2	45.3	54.6	232	76.4	23.6	317
Education level of women						
No formal	45.6	54.3	27	89.9	10.1	110
Primary	56.1	43.9	91	80.8	19.2	183
Secondary	39.2	60.7	219	57.0	43.0	154
College or above	20.3	79.7	47	28.3	71.7	22
Occupation of the women						
Not working	39.9	60.1	296	71.9	28.1	407
Working	46.3	53.6	88	76.9	23.1	62
Birth interval (years)						
0–2	49.1	50.9	29	81.4	18.6	78
≥ 3	40.8	59.2	355	70.8	29.2	391
Known pregnancy complications						
No	44.4	55.6	234	77.4	22.6	346
Yes	36.7	63.3	150	58.9	41.1	123
Receiving ANC by skilled provider						
No	66.1	33.9	68	90.6	9.4	194
Yes	36.1	63.9	316	59.9	40.1	275
Receiving visit by a family planning worker						
No	40.4	59.6	277	76.6	23.4	365
Yes	43.8	56.1	107	58.5	41.5	104
Education level of husbands of the women						
No formal	53.3	46.7	66	82.6	17.3	168
Primary	47.9	52.1	119	77.8	22.2	182
Secondary	40.4	59.6	143	52.3	47.7	84
Higher	15.8	84.2	56	45.8	54.2	35
Religion						
Islam	41.6	58.4	335	73.4	26.6	422
Others	40.0	60.0	49	65.0	35.0	47
Place of residence						
Urban	26.2	73.8	91	56.1	43.9	72
Rural	46.1	53.9	293	75.6	24.4	397
Wealth quintile						
Poorest	69.7	30.3	75	88.9	11.0	158
Poorer	48.7	51.3	79	83.9	16.1	106
Middle	41.3	58.7	84	68.1	31.9	74
Richer	23.7	76.3	83	54.1	45.9	73
Richest	21.6	78.4	62	36.5	63.5	58

^a^Weighted frequency (row percentage)

^b^Total Weighted number

Table 2 provides the results of bivariate and multivariable logistic regression analyses. In Khulna Division, highly educated women had increased likelihood to be delivered by SBAs (COR: 3.3; 95% CI: 1.4–7.9). Women who received ANC by a skilled provider were three times more likely to use SBAs during delivery than the women who did not receive ANC service. The education level of husbands above college level was also a positive predictor for SBA-attended deliveries (COR: 3.3; 95% CI: 1.4–7.9). These patterns were similar in Sylhet.

**Table 2:** Results of logistic regression analyses of deliveries attended by skilled birth attendants (SBAs) with selected background characteristics, in Khulna and Sylhet Division, Bangladesh

Characteristics	SBA Attendance			
	Khulna Division		Sylhet Division	
	COR (95% CI)	AOR (95% CI)	COR (95% CI)	AOR (95% CI)
Current age (years)				
15–24	Ref		Ref	Ref
25–34	1.0 (0.7,1.4)		1.1 (0.7,1.7)	1.2 (0.8,2.0)
35–49	1.5 (0.5,4.4)		0.3* (0.1,0.7)	0.4* (0.2,0.9)
Parity				
1	1.5** (1.1,2.0)	1.3 (0.9,1.8)	1.8* (1.0,3.1)	0.8 (0.4,1.8)
≥ 2	Ref	Ref	Ref	Ref
Education level of women				
No formal	Ref	Ref	Ref	Ref
Primary	0.7 (0.3,1.3)	0.6 (0.3,1.3)	2.1** (1.3,3.6)	1.6 (0.8,3.0)
Secondary	1.3 (0.7,2.4)	0.7 (0.3,1.3)	6.7*** (2.7,16.7)	3.1* (1.3,7.6)
College or above	3.3** (1.4,7.9)	0.7 (0.2,2.2)	22.7*** (7.8,66.1)	8.4** (1.9,36.7)
Occupation of the women				
Not working	1.3 (0.8,2.0)		1.3 (0.7,2.5)	
Working	Ref		Ref	
Birth interval (years)				
0–2	0.7 (0.3,1.6)		0.6 (0.3,1.0)	0.7 (0.3,1.5)
≥ 3	Ref		Ref	Ref
Known pregnancy complications				
No	Ref	Ref	Ref	Ref
Yes	1.4^1^ (1.0,1.9)	1.0 (0.7,1.4)	2.4* (1.0,5.5)	0.9 (0.5,1.6)
Receiving ANC by skilled provider				
No	Ref	Ref	Ref	Ref
Yes	3.5*** (2.0,6.0)	2.5** (1.4,4.6)	6.4*** (2.7,15.6)	3.6*** (2.0,6.5)
Receiving visit by a family planning worker				
No	Ref	Ref	Ref	Ref
Yes	0.9 (0.6,1.3)	0.9 (0.6,1.6)	2.3** (1.4,3.8)	3.0*** (1.6,5.4)
Education level of husbands of the women				
No formal	Ref	Ref	Ref	Ref
Primary	1.2 (0.8,2.0)	0.9 (0.6,1.6)	1.4 (0.8,2.4)	0.6 (0.3,1.4)
Secondary	1.7 (1.0,2.8)	0.8 (0.4,1.4)	4.4** (1.5,12.5)	1.1 (0.4,3.1)
Higher	6.1*** (2.9,13.0)	1.9 (0.6,5.4)	5.6** (2.0,15.6)	0.6 (0.1,3.1)
Religion				
Islam	Ref		Ref	
Others	1.1 (0.5,2.2)		1.5 (0.7,3.0)	
Place of residence				
Urban	2.4*** (1.5,3.9)	1.6 (1.0,2.5)	2.4** (1.3,4.6)	1.4 (0.9,2.2)
Rural	Ref	Ref	Ref	Ref
Wealth quintile				
Poorest	Ref	Ref	Ref	Ref
Poorer	2.4** (1.3,4.6)	2.6** (1.3,5.2)	1.5 (0.6,3.8)	0.9 (0.4,2.0)
Middle	3.3*** (1.7,6.1)	3.0** (1.5,6.0)	3.8* (1.3,10.5)	2.0 (0.9,4.4)
Richer	7.4*** (3.6,14.9)	6.1*** (2.7,13.9)	6.8*** (2.4,19.3)	2.6** (1.4,5.1)
Richest	8.3*** (4.1,16.7)	4.7*** (1.9,11.5)	14.0*** (5.6,34.8)	3.8*** (1.9,7.6)

^1^*p* < 0.2, *p < 0.05, **p < 0.01, ***p < 0.001; *COR* crude odds ratio, *AOR* adjusted odds ratio, *CI* confidence interval

In the results of multivariable logistic regression analyses, the study divisions had two common statistically significant variables, including receiving ANC by a skilled provider and higher wealth quintiles. Sylhet division had only three variables that were not significant for Khulna, which included women with an age of 35–49 years (AOR: 0.4; 95% CI: 0.2–0.9), secondary (AOR: 3.1; 95% CI: 1.3–7.6) or higher education level of women (AOR: 8.4; 95% CI: 1.9–36.7), and receiving visit from an FPW (AOR: 3.0; 95% CI: 1.6–5.4).

## Discussion

In this study, we compared practices, distributions and determinants of delivery attendance in two divisions of Bangladesh. We found that contextual (e.g., receiving ANC by a skilled provider) and socioeconomic factors (e.g., wealth quintiles) significantly influence deliveries by skilled attendants in both regions. Uniquely, in Sylhet, education level of women and visits by an FPW contributed to increased likelihood to be delivered by an SBA. Khulna had higher SBA attendance which was associated with higher proportions of educated women, educated husbands of women, women with ANC services during pregnancy, and richer or richest wealth quintiles in comparison to Sylhet.

Although the main objective of this study was to compare the two divisions with ‘extreme’ SBA attendance in delivery, SBAs are required for all women regardless of age, socioeconomic conditions or geographic locations [4]. The government could utilize the findings from this study to scale up SBA attendance in Khulna division to provide this service to all women. Based on resource limitations and priorities, the government should increase birth attendance awareness programs for women living in Sylhet Division. Of the women who were attended by SBAs in both divisions, the majority were attended by qualified doctors. This may be due to facility delivery of those women, as women who were attended by SBAs were delivered at health facilities where the doctors mainly attended the patients, while in community doctors did not attend women at home [5]. Although the distribution of SBAs in these two divisions was not investigated in this study and there is currently a lack of information about distributions of SBAs in the community level due to unavailability of a centralized database, this could be a potential area of further investigation. The proportion of deliveries attended by CSBAs was very low in both Khulna and Sylhet divisions. The CSBA training program was introduced in 2003 with an aim to provide at least 2 CSBAs in each union(union is the lowest administrative unit of this country) of Bangladesh [21]. From 2003 to 2012, 7000 CSBAs received training, however, lower utilization has been found by several studies from Bangladesh [11, 22]. Policymakers and stakeholders must re-evaluate the effectiveness of the CSBA training program.

In this study, the education level of women had a significant association with SBA attendance in childbirth in Sylhet Division; several other studies from Bangladesh have observed this same trend [10, 11, 13]. Educated women are more aware of the importance of SBAs during the time of childbirth which influences their choices related to care-seeking. Moreover, education is closely linked with wealth status, and it is a consistent predictor of health status although it had no association with SBA-attended deliveries in Khulna [19]. This finding indicates that improvement of education level is crucial to increase the proportion of SBA-attended deliveries; it is also important to accelerate the awareness programs among women with lower education level, potentially through the community health workers or other existing infrastructures. Programs to incentivize retention of girls to complete higher secondary schools exist in Bangladesh [23]; however, these programs must be strengthened to encourage young girls to continue their education up to college, if not beyond.

A small proportion of women received visits from an FPW in both divisions. Though it did not have any significant effects in Khulna, the likelihood of SBA-attended deliveries was lower in Sylhet without visits by an FPW. The government could also explore the effectiveness of FPW for SBA deliveries in Sylhet. In addition to providing contraceptive services, they could counsel women about the importance of SBA-attended deliveries.

Receiving ANC by SBAs results in higher numbers of deliveries by SBAs is also found in other studies [10, 11, 13, 14]. Receiving counseling and information from SBAs during pregnancy could increase the likelihood of being delivered by SBAs. ANC is recommended to identify pregnancy complications [24]. Antenatal counseling has been described as one of the four main pillars of the Safe Motherhood Initiative because of its effectiveness in reducing maternal and neonatal mortality [25]. The government thus should focus on this factor to increase the number of deliveries by SBAs. A significant proportion of women did not receive ANC in Sylhet; more awareness programs are required to increase ANC services in this region. Additionally, a number of studies examined factors associated with ANC utilization in Bangladesh. Those studies also found significant associations between education level and wealth status with ANC utilization [26, 27]. As those factors are similar to the factors associated with SBA-attendance, the Government of Bangladesh could incorporate these findings to increase the percentage of women with ANC utilization and SBA-attendance.

Women from higher wealth quintiles had positive associations with deliveries by skilled attendants in both regions; they were able to afford deliveries by SBAs, as it is difficult to reach health facilities or quality health services in the country for lower income families [10, 12, 13]. In both regions, the higher the wealth quintile - the higher the proportion of deliveries attended by SBAs. We found a lower proportion of women from the two lowest wealth quintiles in Khulna, these inter-divisional and intra-divisional wealth disparities could contribute to this difference in SBA utilization. Although the health care costs in government health facilities are free, a previous study found that hidden costs associated with care-seeking in public hospitals are major impediments to utilize the services for obstetric care [28].This is another area to be intervened upon by the policymakers of the country. There is little published recent data on wealth disparities across divisions of Bangladesh; however, using the same method to estimate the overall wealth status, we found that the proportion of women in the lower two wealth quintiles was higher in Sylhet compared to Khulna. Despite the challenges of interventions aimed at improving wealth status, a review of the BRAC-ICDDR,B Joint Research Project Working Paper Series in Bangladesh found microcredit programs to be effective in improving maternal and child health [29]. The government should continue to implement similar microcredit programs in order to improve the overall socioeconomic status of the country. The wealth status could also have an association with access to transport. People of Khulna region have higher access to mechanical vehicles compared to people of Sylhet region [5]. Transport is an important factor in access to healthcare services; this could be further investigated in relation to access to prenatal visit utilization or skilled delivery assistance.

As the BDHS 2014 found that a substantially lower number of women delivered in health facilities in Sylhet division, it is also important to discuss the significance of facility deliveries in this context [5]. The ‘Three Delays Model’ suggest that delays in ‘recognizing’ the importance of care and ‘reaching’ care could cause adverse outcomes due to delays in ‘receiving’ care from health facilities. Readiness and quality of care infacilities, however, are important to ensure trust to reach health facilities during child birth [30, 31]. Although we did not investigate the quality of care in the health facilities in Sylhet or Khulna regions, earlier reports reveal that the quality of the services provided by health facilities in this country is poor [32]. It is important to provide an ‘enabling environment’ to utilize maternal health services provided by the Government of Bangladesh.

### Limitations and strengths

We were unable to encompass all factors which were observed by other studies due to limitation of the dataset. Associations of complications related to delivery, distance to the nearest hospital, transport issues to take women to health facilities, presence and geographic distributions of SBAs, and cost of SBAs which might be difficult to bear for lower income families were found to be associated with deliveries by SBAs [10–13, 15, 16, 33–35]. Studies from other countries also found these associations with SBA-attended deliveries [36–38].We only analyzed data from survived women, thus we did not examine the determinants of mothers who experienced more adverse impact from delivery complications. This was a cross-sectional dataset from a survey; therefore, causality cannot be established as the conditions may have changed after delivery. Recall bias could affect the recorded responses of participants, as data were collected retrospectively.

Strengths of this study include the generalizability of results for the two divisions of Bangladesh; it covered urban and rural areas of all districts (the second largest administrative unit of the country after division) of the two divisions. Additionally, a large sample size, high response rate, and little missing data of the survey lead to more stable results. This is the only study from Bangladesh which examined and compared two divisions for the practices, distributions and determinants of deliveries attended by SBAs. This investigation will enhance our understanding of the differences in distributions of the factors which influence deliveries by skilled attendants and would shed a new light on a potential region-specific approach to improve the coverage of SBAs in Sylhet division.

## Conclusions

This study compared practices, distributions and determinants of delivery attendance in two divisions of Bangladesh. The government of Bangladesh should address modifiable factors associated with SBA-attendance on a priority basis to achieve the targets of the UN’s SDGs in Sylhet division. The major finding of this study about the differences and association of socioeconomic conditions underscore the significance of improving education level and wealth status in Sylhet. Additionally, providing ANC services by skilled health care providers along with SBA promotion, increasing services of FPW, and implementing awareness programs among the people with lower education level could increase the proportion of deliveries by SBAs. For both Sylhet and Khulna divisions, it is of vital importance to re-evaluate the CSBA training program to increase attendance of SBAs during child birth at the community level.

## Abbreviations

ANC: Antenatal care
AOR: Adjusted odds ratio
ARR: Annual rate of reduction
BDHS: Bangladesh Demographic and Health Survey
CHCP: Community health care provider
CI: Confidence interval
COR: Crude odds ratio
CSBA: Community skilled birth attendants
EA: Enumeration area
FPW: Family planning worker
HPNSDP: Health, population and nutrition sector development program
LB: Live births
MMR: Maternal mortality rate
NMR: Neonatal mortality rate
OR: Odds ratio
SACMO: Sub-assistant community medical officer
SBA: Skilled birth attendants
SDG: Sustainable development goals
TBA: Traditional birth attendant
VIF: Variance inflation factor
WHO: World Health Organization
